# Smyd5 plays pivotal roles in both primitive and definitive hematopoiesis during zebrafish embryogenesis

**DOI:** 10.1038/srep29157

**Published:** 2016-07-05

**Authors:** Tomoaki Fujii, Shin-ichiro Tsunesumi, Hiroshi Sagara, Miyo Munakata, Yoshihiro Hisaki, Takao Sekiya, Yoichi Furukawa, Kazuhiro Sakamoto, Sumiko Watanabe

**Affiliations:** 1 Department of Cancer Genome Research, Sasaki Institute, Sasaki Foundation, Tokyo 101-0062, Japan; 2 Department of Coloproctological Surgery, Juntendo University, Faculty of Medicine, Tokyo 113-8421, Japan; 3 Division of Molecular and Developmental Biology, Institute of Medical Science, The University of Tokyo, Tokyo 108-8639, Japan; 4 Division of Clinical Genome Research, Advanced Clinical Research Center, The University of Tokyo, Tokyo 108-8639, Japan; 5 Fine Morphological Analysis Group Medical Proteomics Laboratory Institute of Medical Science, The University of Tokyo, Tokyo 108-8639, Japan

## Abstract

Methylation of histone tails plays a pivotal role in the regulation of a wide range of biological processes. SET and MYND domain-containing protein (SMYD) is a methyltransferase, five family members of which have been identified in humans. SMYD1, SMYD2, SMYD3, and SMYD4 have been found to play critical roles in carcinogenesis and/or the development of heart and skeletal muscle. However, the physiological functions of SMYD5 remain unknown. To investigate the function of Smyd5 *in vivo*, zebrafish were utilised as a model system. We first examined *smyd5* expression patterns in developing zebrafish embryos. *Smyd5* transcripts were abundantly expressed at early developmental stages and then gradually decreased. *Smyd5* was expressed in all adult tissues examined. Loss-of-function analysis of Smyd5 was then performed in zebrafish embryos using *smyd5* morpholino oligonucleotide (MO). Embryos injected with *smyd5*-MO showed normal gross morphological development, including of heart and skeletal muscle. However, increased expression of both primitive and definitive hematopoietic markers, including *pu.1*, *mpx, l-plastin,* and *cmyb*, were observed. These phenotypes of s*myd5*-MO zebrafish embryos were also observed when we introduced mutations in *smyd5* gene with the CRISPR/Cas9 system. As the expression of myeloid markers was elevated in *smyd5* loss-of-function zebrafish, we propose that Smyd5 plays critical roles in hematopoiesis.

Histone modification constitutes one epigenetic mechanism that plays a critical role in the dynamic regulation of chromatin structure and gene expression, and several enzymes that catalyse histone modifications have been identified^1^. Histone lysine residue methylation contributes both positively and negatively to gene transcription, and a family of histone lysine methyltransferase containing the evolutionally conserved catalytic SET domain has been reported^2^. More than 60 SET domain-containing proteins have been identified in the mammalian genome; among them, the SMYD family, which is comprised of five members in humans, SMYD1–5, has been described^3^,^4^. Members of the SMYD family have been implicated in diverse biological functions in skeletal and cardiac muscle development as well as in cancer progression^5^,^6^,^7^,^8^,^9^. SMYD1, SMYD2, and SMYD3 show histone H3K4 methyltransferase activity^7^,^10^,^11^, as SMYD2 and SMYD3 methylate histones H3K36me2 and K5me1, respectively^12^,^13^. In addition, SMYD2 mediates the methylation of lysine residues of non-histone proteins such as tumour suppressor p53, retinoblastoma (RB), heat shock protein 90 (HSP90), and poly ADP-ribose polymerase (PARP1)^14^,^15^,^16^,^17^. Moreover, SMYD3 also catalyses non-histone proteins, such as vascular endothelial growth factor receptor (VEGFR) and mitogen-activated protein kinase 3/2 (MAPK3/K2)^18^,^19^. Unlike other family members, SMYD5 does not contain a C-terminal tetratricopeptide repeat (TPR) domain^20^. SMYD5 trimethylates H4K20 and negatively regulates inflammatory response genes^21^. However, the physiological function of SMYD5 remains largely unknown.

Zebrafish (Danio rerio) provide an excellent model system with which to study the biological processes of vertebrates. Similar to mammalian models, zebrafish hematopoiesis consists of both primitive and definitive waves^22^. The primitive hematopoiesis wave occurs in the intermediate cell mass (ICM). Blood cell circulation begins around 24 hours post-fertilisation (hpf), at which time, hematopoiesis shifts from ICM to the posterior blood island (PBI)^22^. The definitive wave occurs in the aorta-gonadmesonephros (AGM) around 30 hpf 23. There are three hematopoietic stem cell (HSC) migration and colonisation events beginning around 48 hpf. AGM progenitor cells migrate to the caudal hematopoietic tissue (CHT), an intermediate site of hematopoiesis. Next, lymphocyte differentiation occurs in the thymus. Finally, kidney marrow produces all hematopoietic cell types, which corresponds to bone marrow hematopoiesis in mammals^24^.

Five members of the Smyd family have been identified in zebrafish^25^. In the work described herein, we aimed to determine the physiological function of Smyd5 in zebrafish embryogenesis. Using a morpholino oligonucleotide (MO)-mediated knockdown and CRISPR/Cas9 knockout approach to *smyd5* during zebrafish embryonic development, we found that Smyd5 plays a crucial role in hematopoiesis. These results indicate that Smyd5 represents an epigenetic regulator of hematopoiesis during zebrafish embryogenesis.

## Results

### Expression profile of *smyd5* in zebrafish embryogenesis and adult tissues

**Figure 1:**
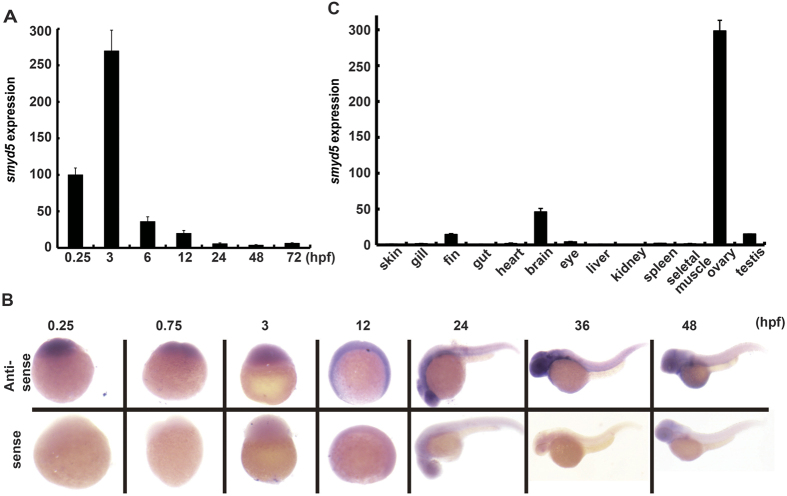
Expression patterns of smyd5 during zebrafish embryogenesis and in adult tissues. (**A**) qRT-PCR analysis was performed using *smyd5* primer sets from RNAs extracted from zebrafish embryos at 0.25, 3, 6, 12, 24, 48, and 72 hpf. (**B**) *In situ* hybridisation of *smyd5* at 0.25, 0.75, 3, 12, 24, 36, and 48 hpf. Lower and upper panels indicate *smyd5* sense control and antisense probes, respectively. (**C**) qRT-PCR analysis of *smyd5* in various adult tissues. Scale bar, 200 μm.

We first examined the expression pattern of *smyd5* during zebrafish embryogenesis by quantitative reverse transcription polymerase chain reaction (qRT-PCR) using RNA extracted from embryos at different developmental stages. *smyd5* was abundantly expressed at early developmental stages but decreased slightly when embryos proceeded in development (Fig. 1A). To examine the spatial and temporal expression patterns of *smyd5* during embryogenesis, a whole-mount *in situ* hybridisation (WISH) assay was performed. *Smyd5* expression was strongly detected from 0.25 to 3 hpf in whole embryos, but it was only weakly observed at 12 hpf. At 24 and 36 hpf, signals were observed only around the eye with stronger intensities at 24 hpf than 36 hpf (Fig. 1B). The distribution of *smyd5* transcripts was also examined in adult tissues by qRT-PCR*. smyd5* transcripts were observed in all tissues examined, but the expression levels were different among tissues (i.e., high in the ovary but relatively weak in the skin, gut, heart, and skeletal muscle) (Fig. 1C).

### Smyd5 is dispensable for heart and skeletal muscle development

**Figure 2:**
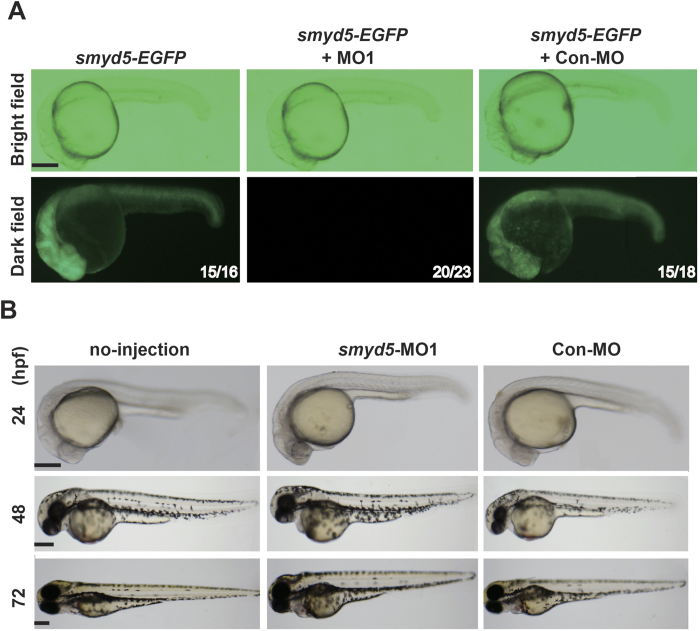
Effect of smyd5 knockdown by MO1 or MO2 in zebrafish embryos. (**A**) Suppression of *smyd5* was examined at 24 hpf in embryos injected with *smyd5*-EGFP mRNA alone, *smyd5*-EGFP mRNA and MO1, and *smyd5*-EGFP mRNA and Con-MO. EGFP signals were examined by fluorescent microscopy (lower panel). Numbers on each panel indicate the number of embryos showing EGFP-positive embryos per total number of embryos. Morphogenesis of zebrafish embryos injected with MO1 (E) or Con-MO (F) at 24, 48, and 72 hpf. Embryos are depicted in the lateral view. Scale bar, 200 μm.

To characterise the physiological functions of *smyd5* during embryogenesis, we used *smyd5*-MOs to knock down the expression of *smyd5* in zebrafish embryos. We designed two MOs, *smyd5*-MO1 and *smyd5*-MO2, which target different regions of the 5′-untranslated region (UTR) of *smyd5*. The efficiency with which *smyd5*-MO1 and *smyd5*-MO2 suppressed *smyd5* expression was tested by co-injection of an expression plasmid encoding the 5′-UTR of *smyd5*; this was followed by enhanced green fluorescent protein (*EGFP*) and control MO (Con-MO), MO1, or MO2. MO and EGFP plasmids were co-injected into zebrafish embryos at the one- to two-cell stages, and EGFP expression was observed at 24 hpf. Co-injection of MO1 but not of Con-MO inhibited the EGFP signal at 24 hpf (Fig. 2A). Similar results were obtained from the injection of MO2 (data not shown), suggesting that MO1 and MO2 efficiently knock down *smyd5*.

We then injected *smyd5*-MO into embryos and examined gross morphological phenotypes. At 24, 48, and 72 hpf, embryos showed no abnormality when observed under binoculate (Fig. 2B). Moreover, zebrafish injected with MO2 did not show visible abnormalities of gross morphogenesis until at least 72 hpf (data not shown).

**Figure 3:**
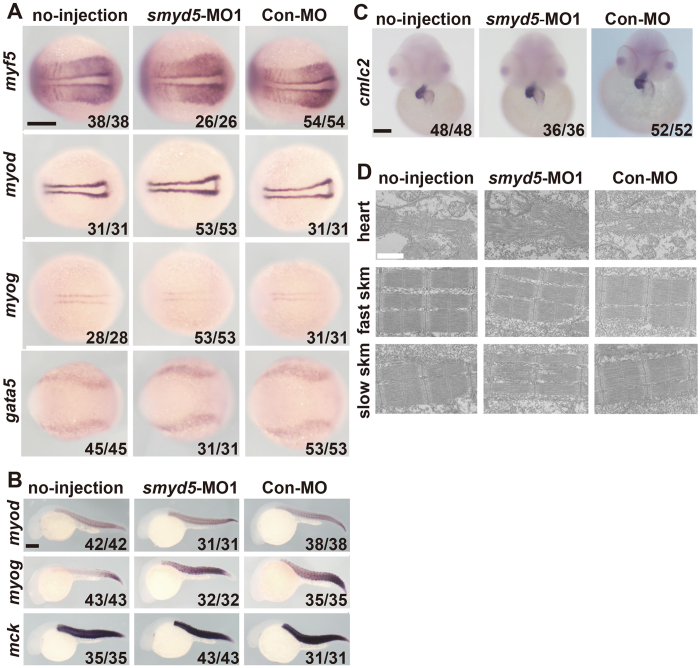
Expression markers for heart and skeletal muscle in smyd5 morphants by WISH and electron microscopic analysis of heart, skeletal muscle, and slow muscle. WISH analysis of skeletal muscle and cardiac chamber markers at 12 (**A**), 24 (**B**), and 48 hpf (**C**). (**D**) Electron microscopic analysis of heat, skeletal muscle, and slow muscle at 48 hpf. (**A**) Expression of *myf5*, *myod*, *myog*, and *gata5* in embryos injected with *smyd5*-MO1, control embryos injected with Con-MO, and those no-injection at 12 hpf. (**B**) Expression of *myod*, *myog*, and *mck* in embryos injected with *smyd5*-MO1, control embryos, and those no-injection at 24 hpf. (**C**) Expression of *cmlc2* in morphants, control embryos, and those no-injection at 48 hpf. Embryos are shown in the dorsal view, anterior towards the left (**A**). Embryos are depicted in the lateral view (**B**) and in the frontal view, dorsal towards the left (**C**). (**D**) Electron micrographs of parasagittal sections through cardiac and somitic muscle cells of embryos injected with smyd5-MO1, and control embryos at 48 hpf. Numbers in the bottom of each panel indicate the number of embryos with the representative phenotype per the total number of examined embryos. Scale bar, 200 μm (black) and 1 μm (white). skm, skeletal muscle.

SMYD family genes play crucial roles in the development of heart and skeletal muscle^10^,^26^,^27^. Therefore, we next examined whether ablation of *smyd5* zebrafish leads to defects in heart and skeletal muscle development by WISH using myogenic and cardiac makers^8^. GATA-binding protein 5 (*gata5*) and cardiac myosin light chain2 (*cmlc2*) are markers for early cardiogenesis and cardiac chamber, respectively^28^,^29^. Myogenic differentiation (*myod*), myogenic factor 5 (*myf5*), and myogenin (*myog*) are myogenic regulatory factors, and muscle creatine kinase (*mck*) is a marker for terminally differentiated skeletal muscle^30^,^31^. At 12 hpf, the expression of *myf5*, *myod*, *myog*, and *gata5* in embryos injected with *smyd5* MO1 was indistinguishable from that in Con-MO-injected embryos or embryos that did not receive an injection (Fig. 3A). These results suggest that Smyd5 is not involved in the early stages of cardiogenesis and myogenesis. Moreover, the intensity of signals and expression patterns of *myod*, *myog*, and *mck* in morphants did not differ from that of control embryos at 24 hpf (Fig. 3B). No difference in the expression of *cmlc2* at 12 hpf was observed between *smyd5*-MO injected morphants and controls at 48 hpf (Fig. 3C). In addition, the structure of sarcomere of heart, fast-and-slow skeletal muscle was indistinguishable between *smyd5*-MO1 injectedembryos and controls at 48 hpf (Fig. 3D). Taken together, these results indicate that Smyd5 is not responsible for the development of cardiac and skeletal muscle.

### Smyd5 is required for primitive myelopoiesis

With the aim of defining the function of Smyd5 in zebrafish, we then focused on the development of hematopoietic cells, which derive from the mesoderm as heart and skeletal muscle. Similar to the case in other vertebrates, zebrafish hematopoiesis consists of two stages, primitive and definitive hematopoiesis^22^. To assess the role of *smyd5* in primitive hematopoiesis, WISH was performed to examine hematopoietic cell markers. *gata1* and *pu.1* are markers for erythroid and myeloid progenitors, respectively^32^,^33^. Hemoglobin beta embryonic 1 (*hbbe1*), lymphocyte cytosolic protein 1 (*l-plastin*), and myeloperoxidase (*mpx*) are markers for erythrocyte, macrophage, and granulocytes, respectively^33^,^34^.

**Figure 4:**
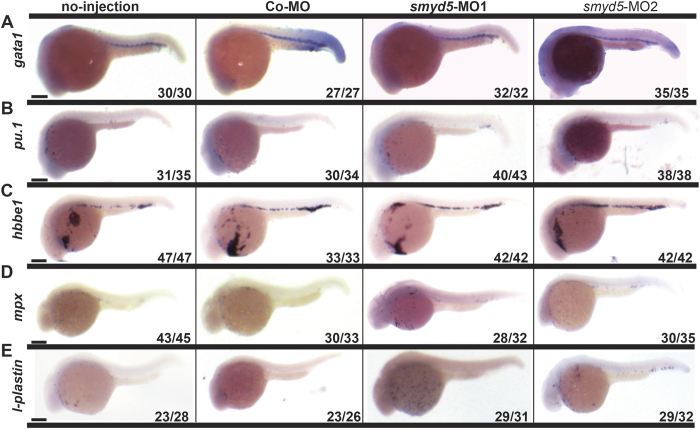
Expression of markers for primitive hematopoietic lineages in smyd5 morphants by WISH. Expression of *gata1* (**A**) and *pu.1* (**B**) in embryos injected with *smyd5*-MO1, *smyd5*-MO2, control embryos injected with Con-MO, and those no-injection at 24 hpf. Expression of *hbbe1* (**C**), *mpx* (**D**), and *l-plastin* (**E**) in embryos injected with *smyd5*-MO1, *smyd5*-MO2, control embryos injected with Con-MO, and those no-injection at 26 (**C**) and 28 hpf (**D,E**). Numbers on each panel indicate the number of embryos showing the representative phenotype per the total number of embryos. Embryos are depicted in the lateral view. Scale bar, 200 μm.

*Smyd5*-MO1 was injected into zebrafish embryos at the two- to four-cell stages, and WISH was performed at each indicated developmental stage. At 24 hpf, expression patterns and signal intensities of *gata1* in embryos injected with *smyd5-*MO1 were comparable to those in control embryos injected with Con-MO or those that did not receive an injection (Fig. 4A). Similarly, at 26 hpf, no difference in *hbbe1* expression was observed between embryos injected with *smyd5*-MO1 and control embryos injected with Con-MO or those that did not receive an injection (Fig. 4B). These data indicate that primitive erythropoiesis is not affected by *smyd5* knockdown. In contrast, although the expression pattern of *pu.1* was comparable to that of control embryos, at 24 hpf, *pu.1* signal intensity was elevated in embryos injected with *smyd5*-MO1 (Fig. 4C). In addition, *mpx* and *l-plastin* expression was increased without perturbation in the expression patterns of embryos injected with *smyd5* MO1 (Fig. 4D,E). Essentially the same results were obtained for *smyd5*-MO2 (Fig. 4A–E).

To test whether the observed results were specific to *smyd5*, *smyd5*-MO1, and *smyd5* mRNA were co-injected into embryos at the two- to four-cell stages. Enhanced expression of c*myb, mpx*, and *l-plastin*, through suppression of *smyd5* expression by *smyd5*-MO1, was reversed by injection of *smyd5* mRNA (Supplemental Fig. 1). These results indicate that Smyd5 negatively regulates the expression of genes related to primitive myelopoiesis in zebrafish.

### Smyd5 is required for definitive myelopoiesis

**Figure 5:**
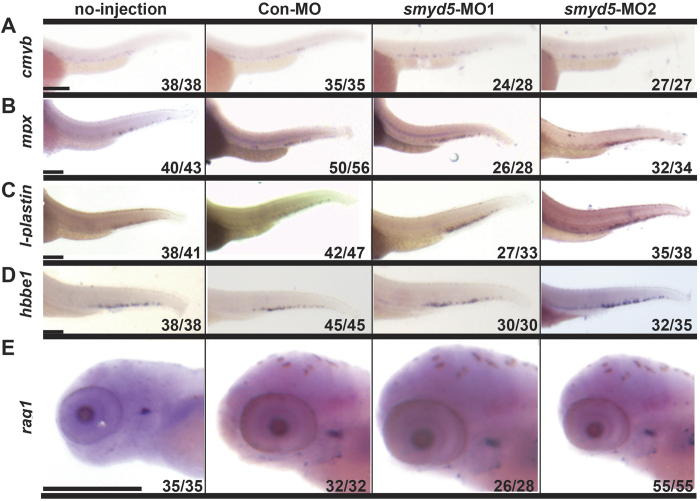
Expression of markers for definitive hematopoietic lineages in smyd5 morphants by WISH. (**A**) Expression of *cmyb* in embryos injected with *smyd5*-MO1, *smyd5*-MO2, control embryos injected with Con-MO, and those no-injection at 30 hpf. Expression of *mpx* (**B**) and *l-plastin* (**C**) in embryos injected with *smyd5*-MO1, *smyd5*-MO2, control embryos injected with Con MO, and those no-injection at 72 hpf. Expression of *hbbe1* (**D**) and *rag1* (**E**) in embryos injected with *smyd5*-MO1, *smyd5*-MO2, control embryos injected with Con-MO, and those no-injection at 96 hpf. The number of embryos with the representative phenotype per the total number of embryos is indicated in each panel. Embryos are depicted in the lateral view. Scale bar, 200 μm.

We then investigated the role of *smyd5* in definitive hematopoiesis by examining the expression of genes related to definitive hematopoiesis. *v-myb avian myeloblastosis viral oncogene homolog* (*c-myb), recombination activating gene 1 (rag1), hbbe1*, *l-plastin*, and *mpx* were examined. By 30 hpf, *c-myb* was expressed in definitive HSCs of AGM^35^. *Rag1* was expressed in lymphocytic lineage^36^. *Smyd5*-MO1 was injected into embryos at the two- to four-cell stages, and WISH was performed using embryos at 30 hpf. *c-myb* expression was increased in embryos injected with *smyd5*-MO1 relative to that of controls, indicating that Smyd5 positively affects definitive hematopoiesis (Fig. 5). In zebrafish, myeloid cells begin to be observed at CHT around 72 hpf, and definitive erythrocytes are detected at PBI at 96 hpf 34,37. At 72 hpf, *l-plastin*- and mpx-expressing cells were observed in CHT in both *smyd5*-MO1-injected and control embryos, and signal intensities were much stronger in *smyd5*-MO1-injected embryos than in controls (Fig. 5B,C). However, at 96 hpf, the *hbbe1* and *rag1* expression of embryos injected with *smyd5-*MO1 was indistinguishable from that of control embryos (Fig. 5D,E), suggesting that Smyd5 does not affect erythropoiesis and lymphopoiesis. Injection of *smyd5*-MO2 resulted in essentially the same phenotypes as that of *smyd5*-MO1(Fig. 5A–E). Co-injection of *smyd5* mRNA rescued the aberrant definitive myelopoieisis induced by *smyd5*-MO1 (Supplemental Fig. 2). Taken together, these findings suggest that SMYD5 negatively regulates definitive myelopoiesis.

**Figure 6:**
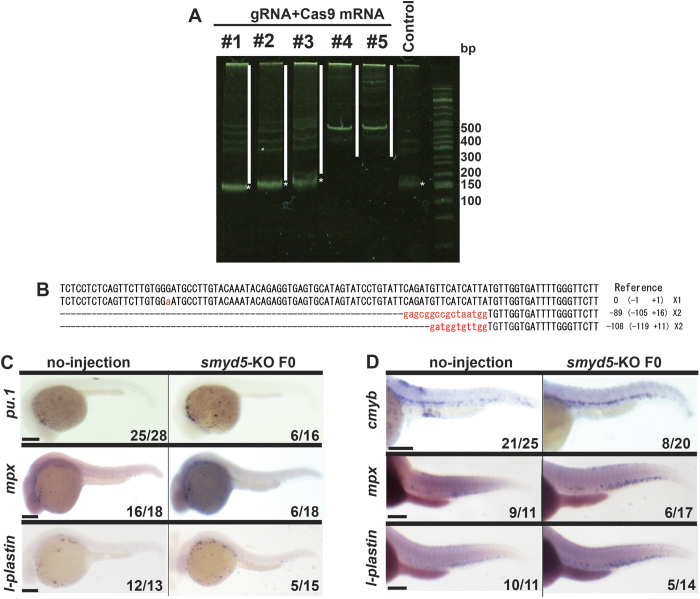
CRISPR/Cas9 targeted mutation of smyd5 phenocopies morpholino knockdown. Identification of embryos with CRISPR/Cas9-mediated insertion and/or deletion (indel) mutations in *smyd5* genomic region by heteroduplex mobility assay (HMA). Heteroduplex (whitelines) and homoduplex (asterisks) DNA band indicate the presence of indel mutant allele, and wild type allele, respectively. Five embryos (#1–#5) were injected with s*myd5* guide RNA (gRNA) and Cas9 mRNA. We detected heteroduplex DNA band in #4 and #5 of embryos. Homoduplex DNA band was detected in #1, #2 and #3 of embryos, which has similar size with that observed in no-injected control. (**B**) Sequences of *smyd5* mutations in #4 embryos. All sequences had indels near the *smyd5* target site of gRNA, which is underlined. Deletions and insertions are indicated by dashe and lowercase red letters, respectively. The number of nucleotides deleted (−) and inserted (+) is indicated to the right with the detection number. (**C,D**) Whole mount *in situ* hybridization of *smyd5*-KO or control embryos. The genes involving primitive myelopoiesis in *smyd5*-KO F0 embryos were examined (**C**). Expression of *pu.1* in *smyd5*-KO F0 embryos, and those no-injection at 24 hpf. Expression of *mpx* and *l-plastin*in in *smyd5*-KO F0 embryos and those no-injection at 28 hpf. Expression of the genes for definitive myelopoiesis in *smyd5*-KO F0 embryos was examined (**D**). Expression of *cmyb* in *smyd5*-KO F0 embryos, and those no-injection at 30 hpf. Expression of *mpx* and *l-plastin*in in *smyd5*-KO F0 embryos, and those no-injection at 72 hpf. Numbers on each panel indicate the number of embryos showing the representative phenotype per the total number of embryos. Embryos are depicted in the lateral view. Scale bar, 200 μm. The English in this document has been checked by at least two professional editors, both native speakers of English. For a certificate, please see: http://www.textcheck.com/certificate/zHsOLC.

To validate the Smyd5 loss-of-function phenotypes, we used CRISPR/Cas9-mediated genome editing system to generate F0 mutants. The guide RNA (gRNA) against *smyd5* genomic (coding) region was designed and injected in combination with Cas9 mRNA into embryos. We confirmed mutations in the target region with heteroduplex mobility assay (HMA) and sequencing analysis. HMA revealed that mutagenesis rates reached 40% (2 of 5 embryos showed only heteroduplex DNA: Fig. 6A). The sequencing analysis showed that all the examined sequences had small insertion and/or deletion near the *smyd5* target loci (Fig. 6B). We then examined the expression pattern of genes, which were modified in *smyd5*-MO injected embryos, by whole mount *in situ* hybridization in embryos injected with *smyd5*-gRNA and Cas9 mRNA (*smyd5*-KO F0). Expression of *pu.1* was increased in *smyd5*-KO F0 embryos than to no-injection controls at 24 hpf (6/16, Fig. 6C). In addition, *mpx* and *l-plastin* signal intensity was elevated in *smyd5*-KO F0 embryos at 28 hpf (6/18 and 5/15, respectively; Fig. 6C). *cmyb* signal intensity was elevated in *smyd5*-KO F0 embryos at 30 hpf (8/20, Fig. 6C). At 72 hpf, *mpx* and *l-plastin* expression was also increased in *smyd5*-KO F0 embryos (6/17 and 5/14, respectively; Fig. 6D). Taken together, the phenotype observed with *Smyd5* knock-down embryos was validated in embryos bearing mutations by CRISPR/Cas9-mediated genome editing system. Therefore, we concluded that Smyd5 regulates primitive and definitive myelopoiesis.

## Discussion

In this report, we showed that zebrafish *smyd5* plays pivotal roles in primitive and definitive hematopoiesis; however, we did not observe an apparent phenotype of the cardiac system or skeletal muscle associated with *smyd5* downregulation during zebrafish development. Previous studies have shown that SMYD1–4 are involved in the development of heart and skeletal muscle in both vertebrates and invertebrates^5^. Deletion of *Smyd1* caused hypoplasia of the right ventricle in mice through disrupted maturation of ventricular cardiomyocytes. Knockdown of *smyd1* also led to malfunction of skeletal and cardiac muscles in zebrafish. Knockdown of *smyd2* in zebrafish impaired cardiac and skeletal muscle development^26^,^27^. We also reported that Smyd3 plays a critical role in cardiogenesis and myogenesis in zebrafish^38^. In addition, muscle-specific depletion of Drosophila *Smyd4* led to the failure of eclosion, resulting in late pupal death^39^. Therefore, it is conceivable that SMYD proteins have an evolutionally conserved function in the development of cardiac and skeletal muscle. Therefore, we examined the expression patterns of various genes specific to cardiac and myogenic markers, and the structure of sarcomere of heart, fast-and-slow skeletal muscle, but no abnormality was observed in *smyd5*-knockdown zebrafish embryos. These results indicate that Smyd5 plays physiological functions that are distinct from those played by the other Smyds. This notion is supported by previous reports describing the roles of SMYDs in cancer progression. All SMYD family members, except SMYD5, have been reported to be involved in the proliferation and survival of a variety of tumors^6^,^7^,^8^. However, SMYD5 was identified to be critical in cancer metastasis in breast cancer cells during lung colonisation^9^.

The structure of SMYD5 also differs from that of other members of the SMYD family. Most SMYD proteins, except SMYD5, possess at least one C-terminal TPR domain, which is critical for its interaction with other proteins^20^. However, the TPR is not present in Smyd5, and SMYD1, SMYD2, and SMYD3 interact with HSP90^5^,^6^,^7^,^8^,^9^,^10^,^11^. HSP90 is a homodimeric, ubiquitous, and essential chaperone involved in a variety of biological processes, including myogenesis and cardiogeneis^5^,^40^. Therefore, a lack of heart and skeletal muscle differentiation may, at least partly, be attributed to the lack of the TPR domain in SMYD5. A proteomics approach to identifying the binding partners of SMYD2, SMYD3, and SMYD5 revealed that SMYD2 and SMYD3 share many interactors, including DNA sliding clamp proliferating cell nuclear antigen (PCNA), replication factors, and mini-chromosome maintenance proteins^11^. These proteins interact with DNA polymerase during the DNA damage response^11^,^20^, suggesting that SMYD2 and SMYD3 share common physiological roles. On the other hand, some proteins involved in DNA repair and chromatin maintenance during the cell cycle have been identified as common interactors of SMYD2, SMYD3, and SMYD5^20^, reflecting both the common and distinct activities of SMYD members through interacting proteins.

We found that Smyd5 plays critical roles in both primitive and definitive myelopoiesis in zebrafish. SMYD5 interacts with nucleophosmin 1 (NPM1)^20^, which regulates myelopoiesis^41^. In addition, NPM1 plays an important role in the regulation of a number of hematopoietic stem cells^42^. NPM1 mutations are commonly observed (~30%) in acute myeloid leukemia (AML), suggesting that SMYD5 participates in myeloid cell differentiation/proliferation through its interaction with NPM1. The role of SMYDs in the hematopoietic system was also observed for other members. SMYD2 regulates the differentiation of regulatory T cells (Tregs) and Th-17 cells^43^, whereas SMYD3 controls inducible Tregs^42^. In both cases, the methyltransferase activities of SMYD for specific target gene loci were suggested to play critical roles^43^,^44^. Based on the current study, we do not have sufficient evidence to prove that SMYD5 exerts its biological function through enzymatic activity, but this is a critical issue that should be addressed in the future. It has been reported that SMYD5 trimethylates histone H4 lysine 20 through its association with nuclear receptor corepressor 1 (NCoR1) complexes, which repress the expression of toll-like receptor 4 (TLR4) target genes^21^. TLR4 signalling promotes granulocyte and macrophage development^45^,^46^. Tlr4 and NCoR are conserved in zebrafish^47^,^48^; based on the current observations, we speculate that enhanced expression of myeloid markers by *smyd5* suppression may be due to suppression of the TLR4 signalling pathway. Interestingly, knockdown of SMYD5 or SMYD3 results in reduced activation of TLR4, but the H4K20me3 mark in TLR4-responsive promoters is largely dependent on SMYD5^21^. These results suggest that SMYD5 and SMYD3 act on different substrates/genomic locations through alternative protein complexes^21^.

Taken together, our current results reveal the important roles of Smyd5 in hematopoiesis and indicate that this activity is specific to Smyd5. These findings will aid in the understanding of the epigenetic regulation underlying hematopoiesis. Future studies will be required to reveal the molecular mechanisms of hematopoiesis through Smyd5.

## Material and Methods

### Maintenance of zebrafish

Zebrafish (Danio rerio) were purchased from a local pet shop and maintained under a 14-h day/10-h night cycle at 28.5˚C. Fertilised eggs were obtained by mating adult fish from outbred colonies soon after the light was turned on. Embryos were staged according to hours post-fertilisation (hpf) and morphological criteria^49^.

### Quantitative reverse-transcription polymerase chain reaction (qRT-PCR) analysis

Total RNA was extracted from embryos and adult tissues using TRIzol® reagent (Invitrogen). cDNA was synthesised using Superscript III reverse transcriptase (Invitrogen) and oligo (dT)15 primers (Invitrogen). Real-time PCR was performed using SYBR Green technology with sets of primers (*smyd5*: forward primer, 5′-ACTTCCTCCTTCACCTCACG-3′, reverse primer, 5′-GTCCAGGTAGCTGATGCAAAT-3′, *ef1a* : forward primer, 5′-CCTTCGTCCCAATTTCAGG-3′, reverse primer, 5′-CCTTGAACCAGCCCATGTT-3′) for *smyd5* on StepOnePlus (Life Technologies). Amounts of transcripts were determined by relative standard curve method, and *ef1a* was used as internal control.

### Microinjection of morpholino-oligonucleotides (MOs), guide RNA and mRNA

All antisense morpholino-oligonucleotides (MOs) were designed and supplied by Gene Tools LCC. Two different MOs, *smyd5*-MO1 (5′-CATTTTAACCTCTAACCTCTCAACC) and *smyd5*-MO2 (5′-CAACCTGACCAATGAGTGTGCGAGA-3′), were designed to hybridise to sequences in the 5′-UTR of *smyd5*. A standard control MO (Con-MO) available from the same manufacturer was used as a control and had no effect on embryonic development under our experimental conditions. MOs were diluted to 1 ng/nl with 1x Danieau buffer, and approximately 3nl was injected into fertilised zebrafish eggs at the one- to two-cell stages using a microinjector (IM-300; Narishige). *Smyd5-*EGFP plasmid was constructed as follows. A fragment containing the 5′-UTR of *smyd5* containing MO target sequences was obtained by RT-PCR using the following primers: 5′-CCGGAATTCTGTTAAAAAAAGAAAGGCGATC-3′ and 5′-CCGCTCGAGGTCATCTACGGGGGCCGC -3′. The fragment was then cloned into pCS2^+^EGFP plasmid and subjected to RNA synthesis. Zebrafish *smyd5* (NM_001004614) cDNA, including the open reading frame (ORF) of *smyd5*, was purchased from GENEWIZ, Inc. Zebrafish *smyd5* cDNA was subcloned into pCS2^+^ to synthesise mRNA. mRNAs were synthesised using m7G(5′)PPP(5′) G (NEB) and SP6 RNA polymerase (Takara). To confirm the effects of *smyd5*-MO knockdown, zebrafish embryos were injected with 100 pg *smyd5-EGFP* mRNA and 300 pg *smyd5*-MO1 or *smyd5*-MO2. Rescue experiments were performed by injection with 300 pg *smyd5* mRNAs and *smyd5*-MO1. A guide RNA (gRNA) was generated as described^50^. Cloning of a gRNA template was initiated by annealing two oligonucleotides (Forward, TAGGCATTCCACAAGAACTGAG and Reverse, AAACCTCAGTTCTTGTGGAATG), and double strand oligonucleotides were ligated into *BsmB*I site of the pT7-gRNA vector (Addgene). To generate the gRNA, template DNA was linearised with *BamH*I, and purified by phenol/chloroform extraction. The gRNA was transcribed *in vitro* by using MEGA short script T7 kit (Thermo Fisher Scientific). Subsequently, 100 pg of gRNA and 150 pg of Cas9 mRNA (SBI, CAS500A-1) were injected into one-cell stage embryos. Embryos were anesthetised on ice and observed under a macro-zoom microscope (MVX10, Olympus).

### Heteroduplex mobility assay (HMA) and sequencing analysis.

To prepare the genomic DNA, embryos at 24 hpf were incubated in 30 μl of 25 mM NaOH, 0.2 mM EDTA at 95 °C for 15 min. Then, 3 μl of 40 mM Tris-HCl (pH8.0) was added to the resultant solution. Genomic fragments at the target sites were amplified by PCR using the following primers: 5′- TCAGGGCAAAGGATTATTCG -3′ and 5′-AAAAAGAACCCAAAATCACCAACA-3′. PCR amplicons were electrophoresed on a 15%polyacrylamide gel containing 10% glycerol. The PCR products were sub-cloned into the pTAC-2 vector (BioDynamics Laboratoey Inc). The plasmid DNAs containing the genomic fragments were prepared from individual colonies, and then, random sequencing was performed.

### Whole-mount *in situ* hybridisation (WISH)

For *in situ* hybridisation, cRNA probes for *gata5*, *cmlc2, mck*, *mylz2*, *myod, myf5*, *myog*, *gata-1*, *pu.1 cmyb hbbe1*, *mpx, l-plastin*, and *rag1* were prepared as follows. cDNAs for these genes (Table 1) were amplified by RT-PCR and products cloned into pcDNA3.1 plasmids (Invitrogen, CA, USA). Digoxigenin (DIG)-labeled RNA probes were transcribed using RNA DIG labelling mix (Roche) and T7 RNA polymerase (Takara). Whole-mount *in situ* hybridisation was performed as described elsewhere^51^. Probe information is presented in Table 1.

### Electron microscopies

Electron microscopy analysis was carried out as previously described with modifications^51^. Sodium cacodylate buffer was used as glutaraldehyde buffer, and the samples were sectioned using Ultracut N and analyzed by HITACHI H-7500 electron microscope.

## Additional Information

**How to cite this article**: Fujii, T. *et al*. Smyd5 plays pivotal roles in both primitive and definitive hematopoiesis during zebrafish embryogenesis. *Sci. Rep.* **6**, 29157; doi: 10.1038/srep29157 (2016).

## Supplementary Material

Supplementary Information

## Figures and Tables

**Table 1:** Genomic regions of probes used for *in situ* hybridisation.

Gene	Probe (nt. no)	Accession number
gata5	397–1147	NM_131235
*cmlc2*	8–639	AF114428
*mylz2*	752–1269	NM_131188
*myod*	313–834	BC056287
*myf5*	131–705	NM_131576
*myog*	391–904	NM_131262
*gata1*	95–1371	NM_131234
*pu.1*	64–742	AF321099
*cmyb*	330–955	NM_131266
*hbbe1*	100–216	BC142787
*mpx*	655–1505	NM_212779.1
*l-plastin*	732–1443	BC062381
*rag1*	2554–3203	U71093
