# Supplementary material for: Smyd5 plays pivotal roles in both primitive and definitive hematopoiesis during zebrafish embryogenesis
Source: Sci Rep. 2016 Jul 5;6:29157. doi: 10.1038/srep29157 (PMC4932602; doi:10.1038/srep29157)
Supplement: Supplementary Information [file srep29157-s1.pdf]

**Supplementary Information**

# Smyd5 plays pivotal roles in both primitive and definitive hematopoiesis during zebrafish embryogenesis

Tomoaki Fujii[1,2,3], Shin-ichiro Tsunesumi[4], Hiroshi Sagara[5], Miyo Munakata[1], Yoshihiro Hisaki[1,3], Takao Sekiya[1],Yoichi Furukawa[4], Kazuhiro Sakamoto[2], and Sumiko Watanabe[3*]

[1] Department of Cancer Genome Research, Sasaki Institute, Sasaki Foundation, Tokyo 101-0062, Japan, [2] Department of Coloproctological Surgery, Juntendo University, Faculty of Medicine, Tokyo 113-8421, Japan, [3]Division of Molecular and Developmental Biology, Institute of Medical Science, The University of Tokyo, Tokyo 108-8639, Japan, [4]Division of Clinical Genome Research, Advanced Clinical Research Center, The University of Tokyo, Tokyo 108-8639, Japan, [5]Fine Morpholigical Analysis Group Medical Proteomics Laboratory Institute of Medical Science , The University of Tokyo, Tokyo 108-8639, Japan

Supplementary Figure S1 and S2

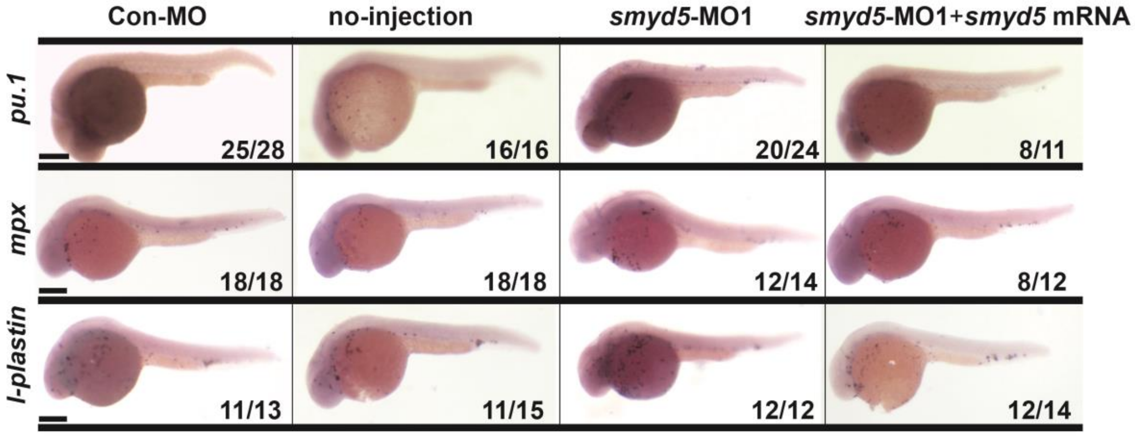


***Supplemental Figure 1. Phenotypic rescue experiments of primitive myelopoiesis in smyd5-knockdown embryos***

Expression of *pu.1* in embryos injected with Con-MO, those no-injection, *smyd5*-MO1, and *smyd5*-MO1 in combination with *smyd5* mRNA at 24 hpf. Expression of *mpx* and *l-plastin*in in embryos injected with Con-MO, those no-injection, *smyd5*-MO1, and *smyd5*-MO1 in combination with *smyd5* mRNA at 28 hpf. Numbers on each panel indicate the number of embryos showing the representative phenotype per the total number of embryos. Embryos are depicted in the lateral view. Scale bar, 200 µm.

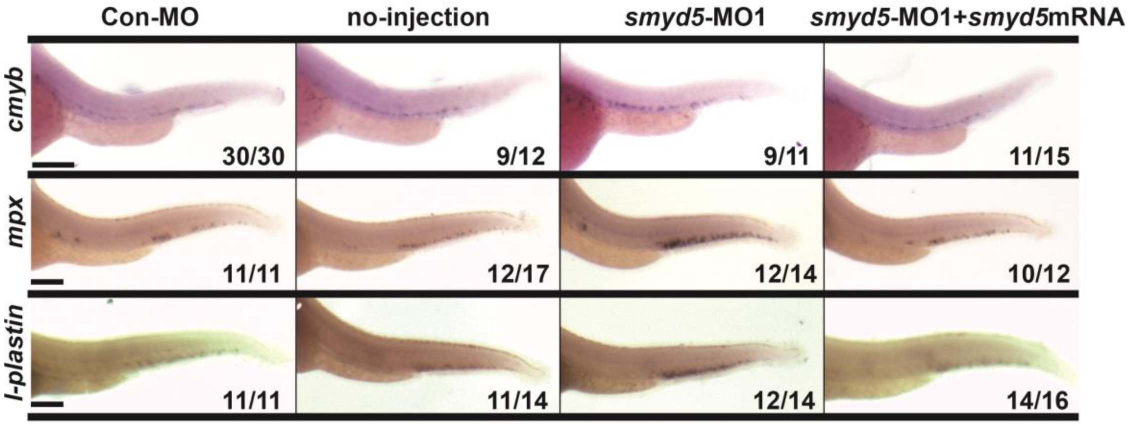


***Supplemental Figure 2. Phenotypic rescue experiments of definitive myelopoiesis in smyd5-knockdown embryos***

Expression of *cmyb* in embryos injected with Con-MO, those no-injection, *smyd5*-MO1, and *smyd5*-MO1 in combination with *smyd5* mRNA at 30 hpf. Expression of *mpx* and *l-plastin*in in embryos injected with Con-MO, those no-injection, *smyd5*-MO1, and *smyd5*-MO1 in combination with *smyd5* mRNA at 72 hpf. Numbers on each panel indicate the number of embryos showing the representative phenotype per the total number of embryos. Embryos are depicted in the lateral view. Scale bar, 200 μm.
